# Influencing viable oral bacteria in the patient's oral cavity and on the dentist’s mask

**DOI:** 10.1186/s12903-026-08628-5

**Published:** 2026-05-28

**Authors:** Madline Priska Gund, Jusef Naim, Josefine Wittig, Janina Lang, Matthias Hannig, Barbara Gärtner, Alexander Halfmann, Gabor Boros, Stefan Rupf

**Affiliations:** 1 Department of Operative Dentistry, Periodontology and Preventive Dentistry, Saarland University Medical Centre, Kirrberger Str. 100, 73D-66421 Homburg, Germany; 2 Institute of Medical Microbiology and Hygiene, Department of Hospital Hygiene, Saarland University Medical Centre, Homburg, Germany; 3 Oral Surgery Clinic, German Armed Forces Central Hospital, Koblenz, Germany; 4 Synoptic Dentistry, Saarland University, Homburg, Germany

**Keywords:** Aerosols, Chlorhexidine, Dentistry, Infection control, Microbiology, Surgical mask

## Abstract

**Background:**

Chlorhexidine (CHX) can lower the bacterial contamination on masks. This study explores how effectively CHX reduces the bacterial spectrum in the patient’s oral cavity and subsequently how this influences the contamination of masks.

**Methods:**

Two intraoral samples were collected prior to any aerosol-generating treatment: the first before a 60-s CHX rinse and the second 10 min after. After dental treatment, the practitioner’s mask was imprinted onto agar plates. After cultivation, a Matrix-Assisted Laser Desorption Ionization Time-of-Flight Mass Spectrometry (MALDI-ToF) was used to identify the colony forming units. The identified species of the intraoral samples and mask imprints were then analysed based upon matches in bacterial species and quantity.

**Results:**

A total of 108 unique patients were included, each receiving one aerosol-producing treatment. The total number of detected bacterial species in the oral cavity before the CHX rinse was 628, after 490. *Staphylococcus aureus* was found twice as often before the CHX rinse. The average amount of colony forming units on the mask’s imprints after the CHX rinse was 15.2. The bacterial species occurred in very similar proportions, with Staphylococcus species making up more than 50%. No species match between the oral samples and mask samples after the CHX rinse occurred most frequently (68.5%), followed by one match (25.9), two matches (3.7%) and three matches (1.9%).

**Conclusions:**

A preprocedural CHX rinse reduces bacterial contamination on masks but has little effect on the spectrum of transmitted microorganisms. Our results suggest that the bacterial spectrum on the mask was not significantly influenced by the preprocedural change in the oral microbiota. Therefore, while CHX enhances clinical hygiene, it does not eliminate the risk of pathogenic transmission.

## Background

Wearing personal protective equipment (PPE) during dental treatments has been mandatory since long before the COVID-19 pandemic. Typically, the PPE in dentistry usually consists of a surgical mask, protective goggles and gloves. Even though these measures became more important during the pandemic, some aspects remained afterwards, particularly the increased focus on hygiene and reducing the risk of infection.

Numerous studies have previously shown that surgical masks contaminate during treatment due to the aerosol generated by a contra-angle handpiece or an ultrasonic scaler [1–5]. Other sources of contamination are contaminated water from the dental unit and airborne contamination [6, 7]. Another study showed that the mask itself holds a potential for contamination after aerosol-producing treatments [8, 9].

Chlorhexidine (CHX) as a pre-procedural mouthrinse has proved to significantly lower the contamination on surgical masks of the practitioner [1]. Another research group revealed the significant reduction of pathogenic microorganisms on the practitioner’s, assistant’s and patient’s chest with the use of a pre-procedural CHX-rinse [10].

Microorganisms originating from the patient ‘s oral cavity have been shown to be present on the mask and to be potentially pathogenic.^2^ Given that bacteria from the patient's oral cavity can transfer to the surgical mask, potentially leading to disease transmission, and that CHX can reduce bacterial contamination on masks, this study aims to investigate how effectively CHX reduces the bacterial spectrum in the patient's oral cavity and, subsequently, how this reduction influences the level of contamination on the practitioner's surgical mask. By understanding this relationship, we aim to decrease the risk of mask contamination, thereby enhancing protective measures for dental staff.

## Methods

### Setting

This prospective study was conducted at Saarland University Medical Centre, Clinic of Operative Dentistry, Periodontology and Preventive Dentistry. The dental units were thoroughly disinfected before and after every treatment (using Celtex® Wipes, Loftex, Bremen, Germany; Incidin® 0,25%, Dräger, Lübeck, Germany). All instruments used during treatment were sterilised. The room temperature was 20–22 °C with a relative humidity of 40–60%.

### Treatments

Following aerosol-producing treatments were included: professional dental cleanings, periodontal treatments, filling therapies and endodontic treatments (trepanation only). The professional dental cleaning consisted of a supragingival cleaning with an ultrasonic scaler (Kavo® PiezoLED Scaler Handpiece), manual instruments and a polish afterwards. Periodontal treatment consisted of a subgingival cleaning with an ultrasonic scaler and manual instruments of infected periodontal pockets. The filling therapies and endodontic treatments included high speed preparation of carious tooth substance and composite for restoration. During the caries excavation a rubber dam was used if the cavitation was located supragingival, for the finishing of the filling it was removed again. The trepanation for endodontic treatment was always performed without rubber dam.

During all treatments the dental water used for cooling was evacuated by a conventional dental suction (CDS) using a cannula of 3.3 mm in diameter (suction flow 1.1 l/s) and a high-volume evacuation (HVE, tube of 8.0 mm in diameter, suction flow 6.0 l/s). The CDS was positioned lingually from the lower central incisors, the HVE next to the aerosol source.

### Ethics approval and consent to participate

The study was conducted in accordance with the Declaration of Helsinki. Adult patients without known infectious diseases were included. The use of antibiotics within six months prior to treatment resulted in exclusion from the study. Written informed consent was obtained from all participants prior to inclusion. All samples were anonymized. Ethical approval for the study was obtained from the Ethics Committee of the Saarland Medical Association (Vote No. 195/22).

### Subjects

**Fig. 1:**
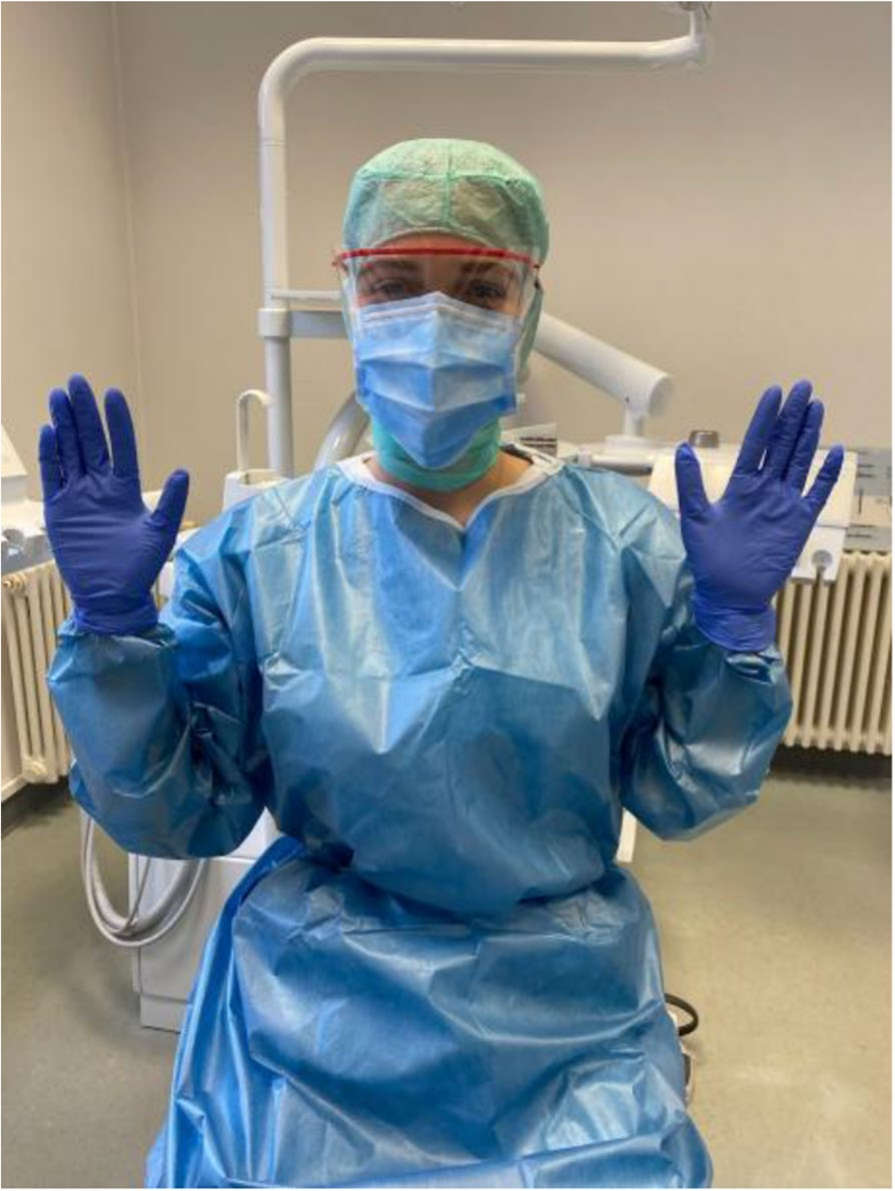
Personal protection equipment: surgical mask, FFP2 mask, examination gloves, protective eyewear, surgical cap, protective gown

The treatments were performed by a total of 7 second-year clinical dental students. They received a detailed instruction in advance. To minimise variations in aerosol diffusion, all students received standardised theoretical and practical training on the correct use and positioning of the HVE. Before applying personal protective equipment (PPE) a hygienic hand disinfection was performed. They wore surgical masks (tie-band medical surgical mask type II, Mölnlycke Health Care, Düsseldorf, Germany), FFP2 masks underneath the surgical masks to prevent a cross-contamination by the subject (Particel Filtering Hals Mask, Shunmei Medical Co., LTD., Shenzhen, China), examination gloves (nitrile powder-free gloves: Jove®, Hebei Titans Hongsen Medical Technology Co., LTD., Hebei, China), protective eyewear (Safeview® eyewear, Halyard, Neunkirchen, Germany), surgical caps (Astro Surgical Cap, FarStar medical GmbH, Barsbüttel, Germany) and a protective gown (Simani Industrie s.r.l., Gallicano, Germany). It was mandatory not to touch the outer surface of the surgical masks. The sampling took place from May 2023 to November 2023 (Fig. 1).

### Sampling

Before starting treatment, an intraoral sample was taken with a flocked nylon fibre tip (Eswab Autom. LQ Amies Regular Tampon Nylon – Flockenfaser, Copan Italia SpA, Italia). The tip was passed along the mucogingival border from the terminal lower right molar buccally to the incisive and lingually back, as well as along the floor of the mouth. It was then stored in 1 ml of Liquid Amies transport medium. Immediately afterwards the patient rinsed with 0.2% CHX (Dynexidin® Forte 0.2%, Chlorhexidinbis (D-gluconat), chemische Fabrik Kreussler & Co. GmbH, Germany) for 60 s. After ten minutes the second sample was taken from the patients mouth likewise to the first one. During those ten minutes the dental assessment took place, without aerosol producing instruments.

**Fig. 2:**
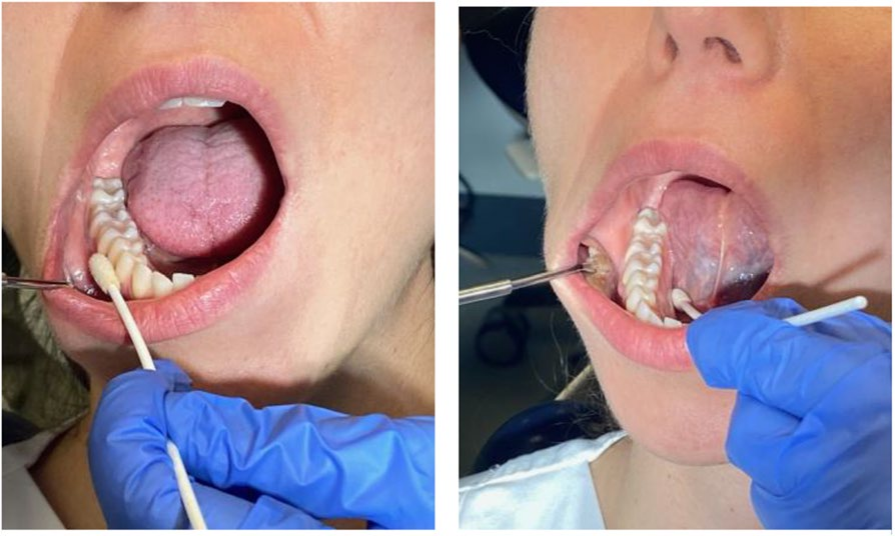
Taking the intraoral sample on the border of the teeth and gingiva

After treatment the surgical mask was removed from the subject by an assistant wearing unused examination gloves without touching the outer surface of the mask. Right away the mask was imprinted onto two agar plates: Columbia (Columbia III Agar with 5% Sheep Blood, Becton Dickinson GmbH, Heidelberg, Germany) and Chocolate agar (Blood Agar No. 2 Base, Becton Dickinson GmbH, Heidelberg, Germany) for ten seconds each (Fig. 2).

### Microbial cultivation

**Fig. 3:**
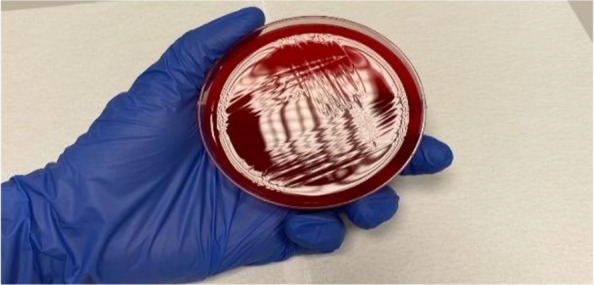
The triple-streak plating method

For the cultivation of the intraoral samples the triple-streak plating method was used. First a spread of Amies medium was applied to the agar plate by the nylon fibre tip and spread with an inoculation loop (Sarstedt, Nümbrecht, Germany) to distance individual bacteria from another. By using a second sterile inoculation loop, that was passing through the first streak two to three times, a second streak was formed. This was repeated for a third strike (Fig. 3).

Afterwards the agar plates were stored in an incubation container for gas generating systems (AnaeroPack Rectangular Jar, Mitsubishi Gas Chemical Company, ING., Tokyo, Japan). During the incubation at 35 °C ± 2 °C for 48 h a gas pack (GasPak™ EZ Container System, Becton Dickson GmbH, Heidelberg, Germany) was applied to assure a carbon dioxide enriched environment.

### Quantitative bacterial analysis

Colony forming units (CFU) were counted using a colony counter (schuett-biotec GmbH, Göttingen, Germany) for mask samples.

The quantitative distribution for the intraoral samples was divided into scores on the basis of the triple streak plating method: Score 0 = no contamination, Score 1 = first streak is colonised with bacteria (colony forming units < 150), Score 2 = first and second streak is colonised with bacteria, Score 3 = all three streaks are colonised.

### Qualitative bacterial analysis

**Fig. 4:**
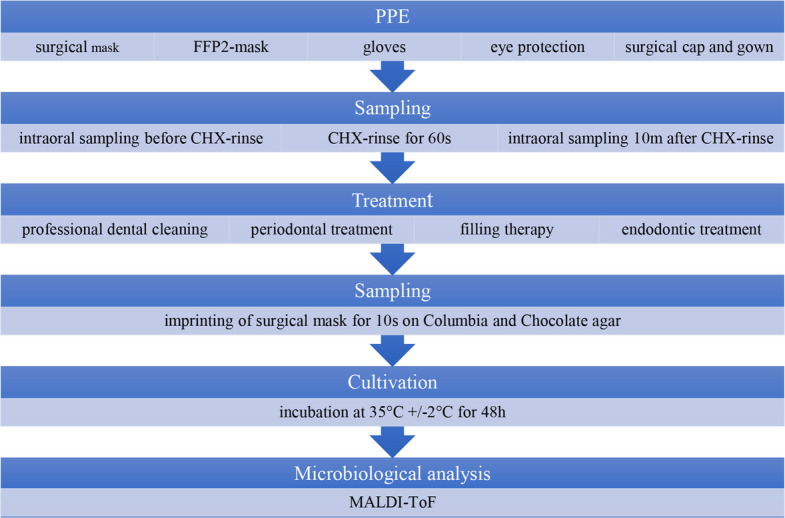
Flowchart of the study

A Matrix-Assisted Laser Desorption Ionization Time-of-Flight Mass Spectrometry (MALDI-ToF Biotyper™ MBT™ smart, Bruker, Daltonik GmbH, Bremen, Germany) was used to identify the colony forming units after phenotypic distinction. The colonies were transferred onto a stainless-steel target (MSP 96 spot target, Bruker Daltonik GmbH, Bremen, Germany). 1 μl of formic acid (AppliChem GmbH, Darmstadt, Germany) was applied on the occupied targets and after being fully dried 1 μl of matrix (Bruker HCCA = α-Cyano-4-hydroxycinnamic acid, Bruker Daltonik GmbH, Bremen, Germany) was added. Then MALDI-ToF started the analysis, identifying the species. If it was not successful, the measuring point of the laser was manually adjusted until the species was identified. In case of a spectrum not being able to be assigned to a known species, it was listed as “unidentified” (Fig. 4).

### Controls

A simulation of aerosol-producing dental treatments (trepanation, cavity preparation, filling therapy) under a similar setting with identical PPE was performed on a phantom head with five unused surgical masks (*n* = 5) for 120 min each. This was to exclude the possibility of contamination by the practitioner itself.

### Statistics

The Quantity of bacteria of both intraoral samples and the mask imprints were compared using the Wilcoxon Signed-Rank Test. Qualitative results of bacteria in the intraoral samples before and after rinsing were compared using Spearman’s Rho Calculator.

The detection frequency of the most frequently occurring bacteria was analysed with the help of the Friedman Test for Repeated Measures and the Wilcoxon Signed-Rank Test.

For the comparison of matching species of the intraoral samples after rinsing and the mask imprints between the different treatment modalities the Kruskal–Wallis test was used (*p* < 0.05).

## Results

### Controls

No microbial growth was detected on the control samples after 48 h of cultivation.

### Bacterial load and quantitative analysis

**Fig. 5:**
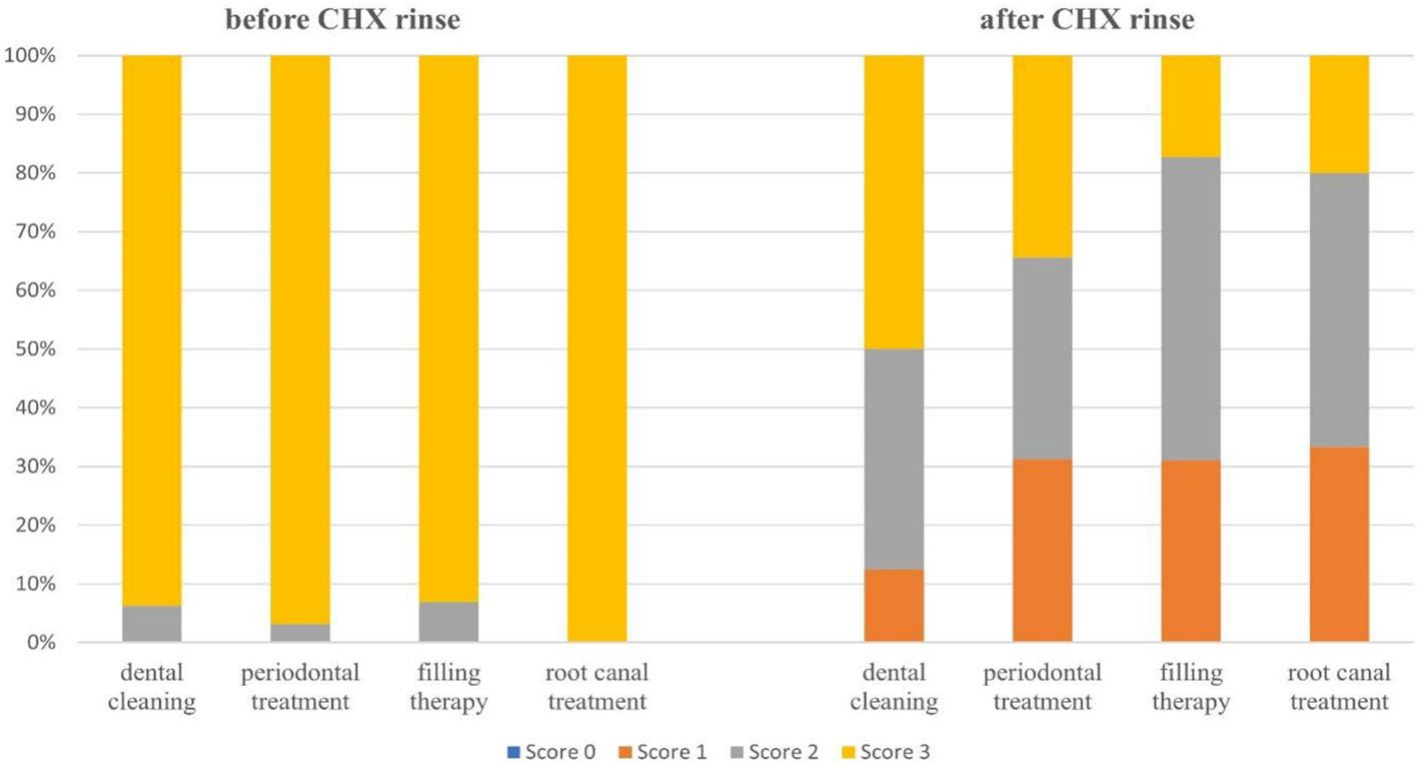
Overview of the different scores of intraoral samples before and after a CHX rinse in percent (*n* = 108)

In total, 108 aerosol-producing dental treatments with an average duration of 139 min were included: 32 professional dental cleanings, 32 periodontal treatments, 29 filling therapies and 15 endodontic treatments. Each patient was included only once (Fig. 5).

On the intraoral samples before the CHX rinse Score 3 was the most common result with more than 90% of the time. After the CHX rinse Score 3 appeared less than 50% of the time.

Score 2 occurred less than 10% of times before the CHX rinse and quadrupled after the CHX-rinse.

Score 1 did not appear at all before the CHX rinse, after the CHX rinse it appeared around a third of the time. Score 0 did not occur at all on intraoral samples.

The total number of CFU detected on all masks was 1642, corresponding to a mean value of 15.2 CFU per mask.

### Bacterial composition and qualitative distribution

**Fig. 6:**
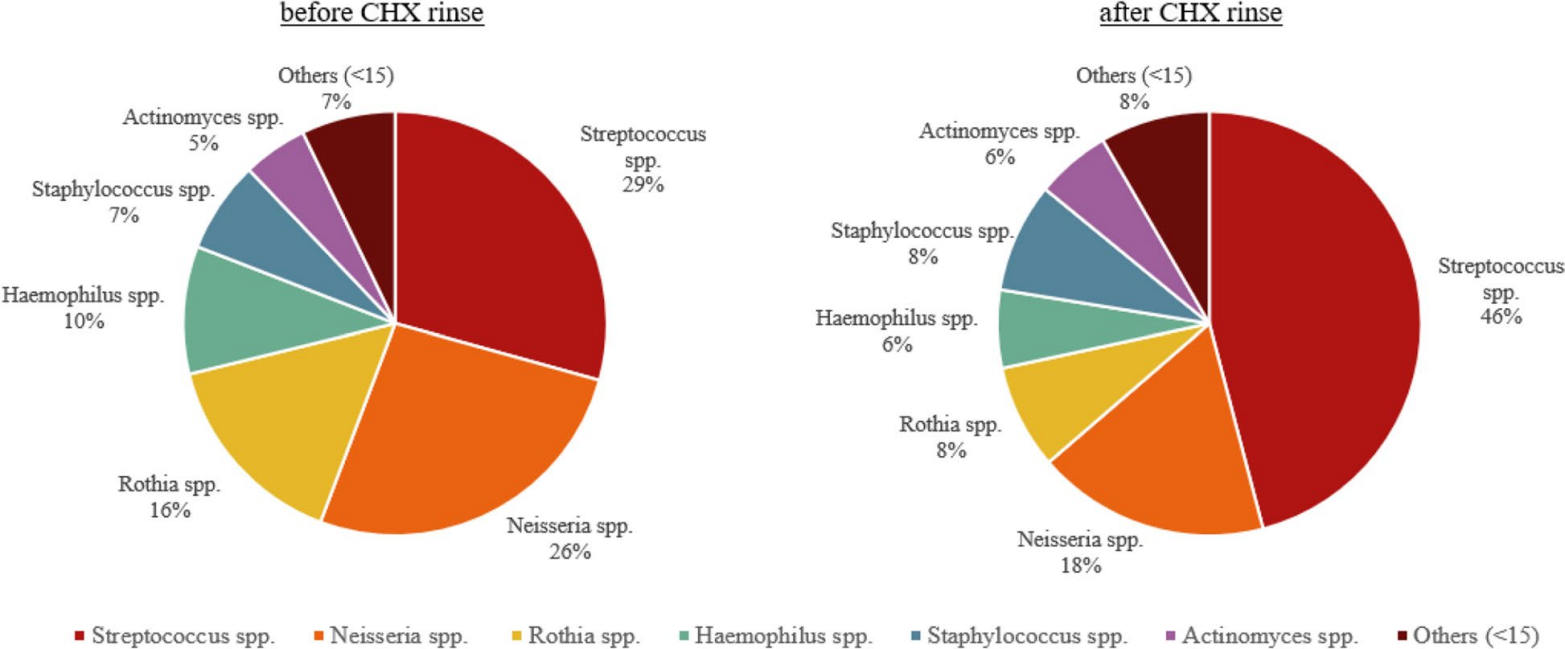
Genus distribution in percent of the oral samples before and after 60 s of CHX rinse of all four treatment modalities (*n* = 108)

**Fig. 7:**
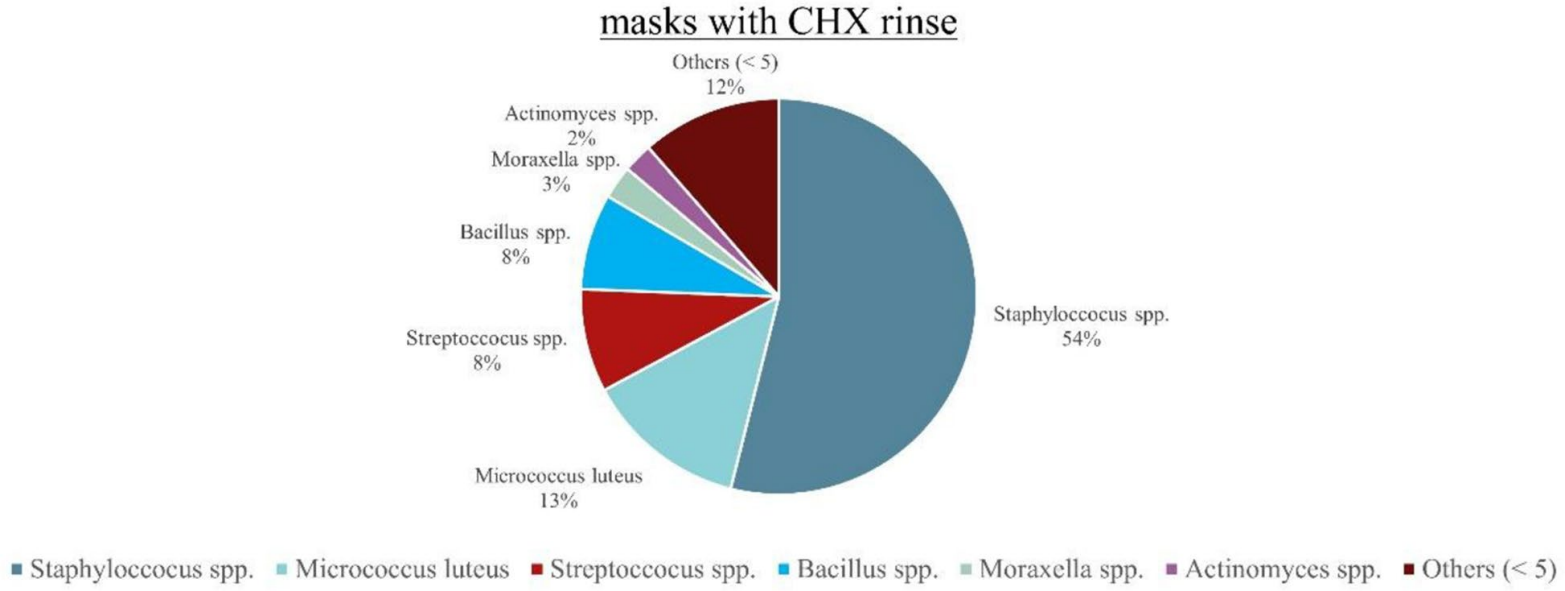
Genus distribution in percent on the masks with prior CHX rinse (*n* = 108)

Regarding the bacterial diversity, the total number of detected bacterial species in the oral cavity was 628 before the CHX rinse and 490 after the rinse (Figs. 6 and 7).

Comparing the oral samples, before the CHX rinse Streptococcus species (spp.) makes up 29%, Neisseria spp. 26% and Rothia spp. 16%. After the CHX rinse Streptococcus (Strep.) spp. makes up 46%, Neisseria spp. 18% and Rothia spp. 8%. In both oral samples Streptococcus species and Neisseria species make up the majority. *Staphylococcus aureus* was found twice as often before the CHX rinse compared to the samples after the rinse.

**Fig. 8:**
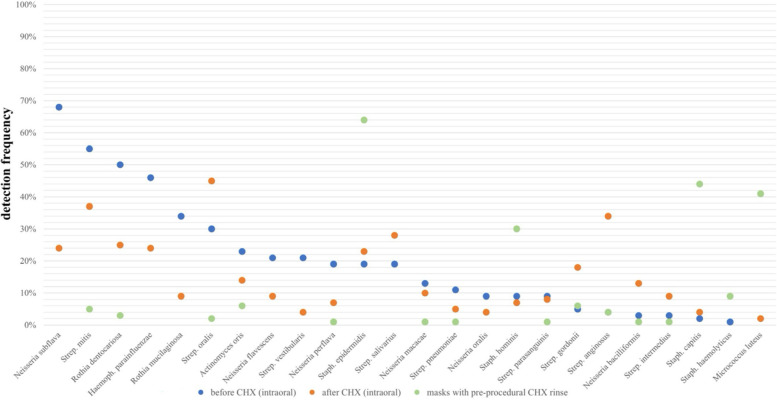
Detection frequency in percent of the intraoral sample before and after a CHX rinse and on mask with a 60 s CHX rinse before treatment

On the mask imprints Streptococcus spp. constitutes only 8%. However, Staphylococcus (Staph.) spp. makes up 54% and Micrococcus luteus 13% (Fig. 8).

The detection frequency shows the percentage of the most frequently occurring microorganisms on the oral samples and the mask imprints with a pre-procedural CHX rinse. On the oral samples before CHX rinsing Neisseria subflava (68%), Strep. mitis (55%) and Rothia dentocariosa (50%) were found most frequently. After the CHX rinse Strep. oralis (45%), Strep. mitis (37%) and Strep. anginosus (34%) showed the most frequency.

On the mask imprints with a 60 s CHX rinse before treatment Staph. epidermidis (64%) was detected the most, followed by Staph. capitis (44%), Micrococcus luteus (41%) and Staph. hominis (30%).

### Matching species

**Fig. 9:**
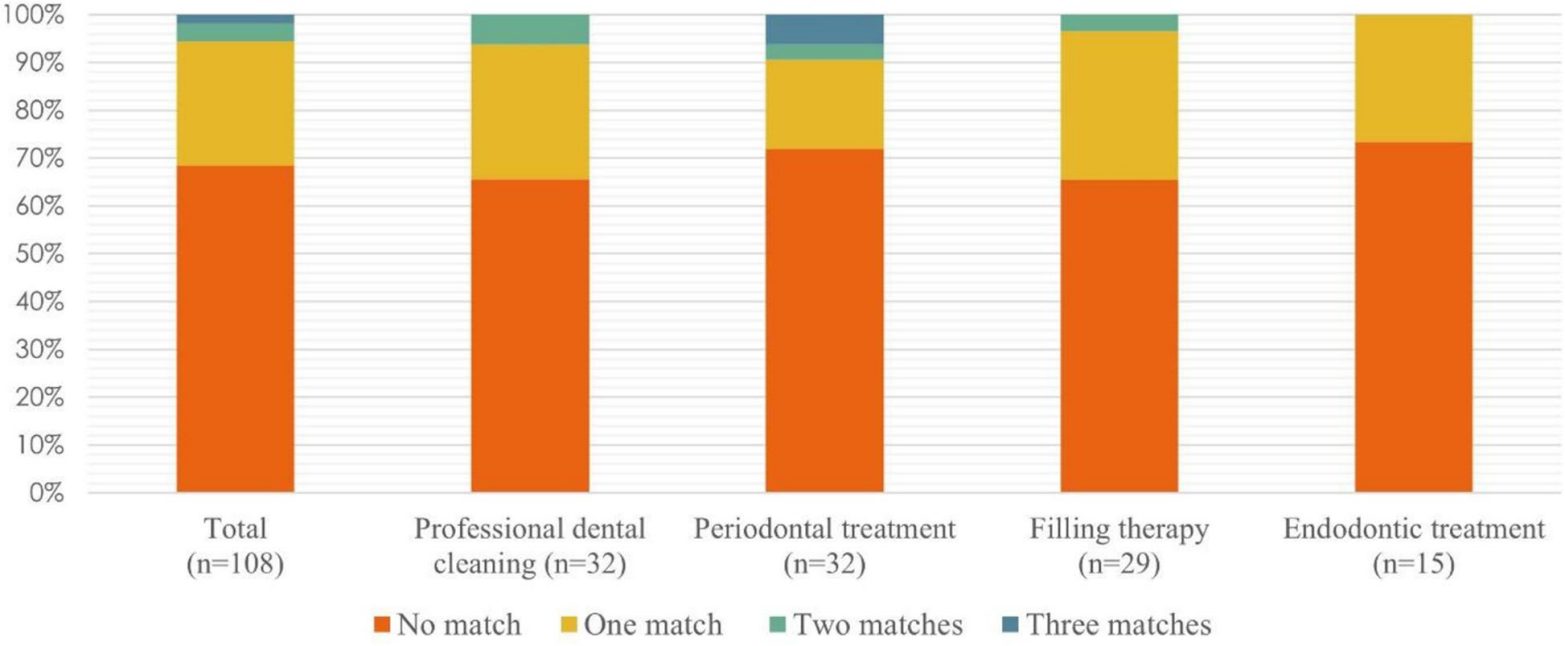
Number of matching species on the intraoral samples after CHX rinse and the mask samples of the four treatment modalities in percent

For the total of all four treatment modalities the number of matching species was zero in most cases (68.5%), followed by one match (25.9%). Two matching species occurred in 3.7%, three matching species were found in 1.9% of all samples. The different treatment modalities showed only minor differences in the distribution. Three matching species only occurred in periodontal treatments (Fig. 9).

### Statistical analysis

The analysis of the bacterial quantity before and after the CHX rinse as well as the mask samples (scores in intraoral samples and CFU on mask imprints) showed a statistically significant difference (*p* < 0.00001).

Bacterial composition (quality): In contrast, the qualitative analysis showed no such fundamental shift. The comparison of the genus distribution before and after the CHX rinse showed no statistically significant difference (*p* = 0.078).

For the detection frequency across the three sample groups (the intraoral samples before and after the rinse and the mask samples) a statistically significant difference was observed (*p* = 0.006). However, the comparison of only the intraoral samples at species level before and after rinsing showed no statistically significant difference (*p* = 0.197). Likewise, the intraoral samples after the CHX rinse and the masks samples (*p* = 0.087).

No statistically significant difference could be determined for the total of matching species of the oral cavity and the mask (*p* = 0.8481).

## Discussion

To the best of our knowledge, this is the first study to compare both the intraoral bacteria of a patient’s mouth before and after a CHX-rinse as well as the contamination of the mask during an aerosol-producing dental treatment afterwards in context. In total, 108 aerosol-producing dental treatments with an average duration of 139 min were included: 32 professional dental cleanings, 32 periodontal treatments, 29 filling therapies and 15 endodontic treatments. A central finding of this study is that while the preprocedural CHX rinse led to a significant reduction in the absolute bacterial load (CFU, Scores), the overall bacterial spectrum remained largely unaffected. In the following, the results are compared to historical control data from a previous study that used identical methodology but without a preprocedural mouth rinse. Both studies applied similar methods, used the same materials and proceeded in identical framework conditions. It is important to note that this a cross-study comparison rather than a single controlled experiment.

The quantitative results of the mask imprints after CHX rinsing (*n* = 108) were compared to the findings of a prior study performed without a preprocedural CHX rinse (historical control, *n* = 126). The number of detected microorganisms on the masks in the historical control group was 417, on the masks with a CHX rinse before treatment it was 332. Therefore, resulting in an average of 3.3 detected microorganisms per mask imprint without a CHX rinse and 3.1 on mask imprints with a CHX rinse prior to treatment (*p* = 0.297). The total of colony forming units (CFU) on the control group was 2934, after the CHX rinse it was only 1642, meaning an average of 23.3 CFU on mask imprints without a CHX rinse and 15.2 CFU on mask imprints with a CHX rinse. The results line up with the literature describing the effectiveness of CHX for reducing the bacterial load in the oral cavity and therefore the contamination risk for the practitioner [11, 12]. However, CHX alone cannot completely prevent contamination of the mask, so the risk of transmission remains.

Furthermore, a comparison between the detection frequencies observed in the previous study and the current CHX group revealed similar patterns. Both cohorts showed the highest detections numbers with Staph. epidermidis (67%), Micrococcus luteus (46%) Staph. hominis (40%), Staph. capitis (39%). Making evident that even though the amount of microorganism can be reduced by CHX, the spectrum of transmitted bacteria remains the same.

Looking at the relative number of matching species, there is a visible decrease on the mask samples with prior CHX rinse. One matching species is found in 25.9% of cases with a CHX rinse, without a rinse it is 26.2%. In 3.7% of cases, two matches occurred, whereas this was the case in 11.9% of cases in the control group. Three matches were found 1.9% in treatments with the CHX rinse and 3.97% without a rinse *p* = 0.043). The results line up with the literature describing the effectiveness of CHX for reducing the bacterial load in the oral cavity and therefore the contamination risk for the practitioner [11, 12].

The bacteria detected on the masks correspond to those described in literature for contamination of PPE [1, 2, 4, 8]. Furthermore, the bacteria found in the intraoral samples match those usually described for the oral cavity [13–15]. A high bacterial count before the CHX rinse in the intraoral samples was to be expected, as the human oral microbiome includes an estimate of 700–800 different species of prokaryotes. This gives an approximate number of viable bacteria of 10^8^ bacteria per millilitre of saliva [9].

The bacteria discovered in this study correspond to those of the natural oral microbiome. It should be emphasised that only healthy patients without infectious diseases were included in this study. In a normal treatment environment, obligate pathogenic bacteria can therefore occur and present a significant risk of infection for the practitioner and other staff. Hence, a transmission of potentially pathogenic bacteria must not be excluded. However, the treatments were executed by dental students who took an average of 139 min per procedure. In everyday clinical practice it cannot be assumed that an experienced dentist needs comparably long for a similar treatment, therefore lowering the time of exposure.

Even with only the participation of healthy patients, some facultatively pathogenic bacteria were still found and will be further discussed:

Staphylococcus epidermidis was most frequently detected on the masks, regardless of a prior mouth rinse with CHX or not. It belongs to the group of coagulase-negative staphylococci and is a facultative, often multiresistant pathogen pathogen posing [2]. The opportunistically pathogenic bacterium Micrococcus luteus [16] occurred the second most common at the genus level on the mask samples.

The intraoral samples showed a reduction of Rothia spp., Neisseria spp., and Haemophilus spp. CHX is a broad-spectrum disinfectant being efficient against Gram-positive, Gram-negative bacteria, fungi and viruses [17].

Overall, the intraoral samples before and after the CHX rinse showed a similar bacterial distribution, while the bacteria on the masks often differed. This indicates that only a small proportion of bacteria was transmitted during treatment. It can also be attributed to the fact that many species cannot be cultivated. To cultivate those bacteria that are more demanding, special culture media would have been needed.

The total amount of bacteria in the oral samples is also to be critically considered, as the sample was only taken in one quadrant while the treatment includes the whole oral cavity. The sampling of the mask may have led to a narrowing of the bacterial diversity as the entire surface of the mask was never pressed onto the agar plates, only the centre of it. Moreover, some species cannot survive on the mask as their ideal habitat is in the oral cavity.

Many genera have a similar appearance and are therefore difficult to distinguish visually. This might explain low numbers of matching species, as different species were potentially recognised as the same species. Taking Strep. mitis and Strep. oralis, both are viridians Streptococci and phenotypically very similar [18]. This could have been limited by identifying more than one colony with the same morphology. Since this is a proof of principle study, the additional effort would have made little difference.

Furthermore, the MALDI-TOF MS analysis identifies bacteria upon the different phenotypes specified beforehand, possibly lowering the bacterial spectrum as well. This means that there are probably more viable bacteria on the mask with an origin of the oral cavity than discovered.

The use of polymerase chain reaction (PCR) would have not been a useful alternative, as this study is about viable bacteria that can be transmitted causing potential infections. It would have made an enormous methodologically effort without benefit. Regarding the topic in question, the chosen method is the most sensible one as it the most reliable in differentiating the colonies.

Additionally, the treatments were performed by a group of 7 dental students in their second clinical year. Although they received a standardised training on HVE and suction protocols to minimise variations in aerosol diffusion, individual differences may have influenced the results.

Although there was a statistically significant difference in the quantity between the three samples (before CHX rinse, after the rinse and the mask imprints), the quality of bacteria before and after the rinse did not show a significant difference.

Interestingly, the detection frequency showed a statistically significant difference in the overall comparison. However, no significant difference was found when comparing only the intraoral samples or when comparing the intraoral sample after the rinse with the mask imprint.

The matching species showed no statistically significant difference. Nevertheless, in full-mouth treatments such as professional dental cleaning and periodontal therapy, an increased trend of matching species can be observed, which is consistent with the findings of the previous study.

In conclusion, a comparison of the results with the control group shows a similar spectrum of bacteria on the mask even when rinsing with CHX before the dental treatment.

Previous studies have also demonstrated that the bacterial spectrum on the mask with a pre-procedural rinsing with CHX and without is very similar [1].

This is the first study to analyse the changes in the oral cavity compared to the changes on the mask after a pre-procedural mouth rinse.

Within the limitations of this study, no clear association was observed between the reduction of oral microbiota and the qualitative bacterial spectrum detected on the masks.

The transmission of facultative and obligate bacteria may still occur.

The microbial spectrum on the mask is essentially determined by the transmission path. For example, not every bacterium is viable on the mask. Of course, as mentioned above, methodological limitations must also be taken into account. The study makes it clear once again that contamination of the mask, and therefore also of the personal protective equipment, can only be reduced, but not prevented in any way. Furthermore, it is not possible to influence the bacterial spectrum on the mask by changing the patient's oral microbiota prior to treatment.

It is therefore essential to take a detailed medical history before treatment to identify possible risk factors and minimise the risk of infection for the practitioner and the environment. However, this may be incomplete, and the patient may be carrying unknown diseases.

It must be assumed at all times that aerosols harbour a certain risk of infection, and this must be kept to a minimum by taking appropriate measures beyond a preprocedural CHX mouth wash. It should also not be overlooked that the mask itself after dental aerosol-producing treatments can become a source of transmission and represent a danger despite all the measures taken. This is particularly important in the case of existing global migration flows and the transfer of microorganisms worldwide.

## Conclusions

This study confirms that a preprocedural CHX rinse reduces bacterial contamination on masks but has little effect on the spectrum of transmitted microorganisms. The reduction of oral bacteria through a CHX rinse did not correlate with a corresponding change in the transmitted bacterial spectrum on the practitioner’s mask in our study. Therefore, while CHX enhances clinical hygiene, it does not eliminate the risk of pathogenic transmission, highlighting the need for strict infection control measures beyond mouth rinsing alone. CHX should complement rather than replace existing protective protocols. Additionally, masks may become secondary contamination sources, emphasizing the importance of continuous protective measures.

## Data Availability

The dataset used and analysed during the current study is available from the corresponding author upon reasonable request.
